# Is chest imaging relevant in diagnosing acute respiratory distress syndrome in polytrauma patients? A population-based cohort study

**DOI:** 10.1007/s00068-019-01204-3

**Published:** 2019-08-10

**Authors:** Karlijn Julia Patricia van Wessem, Luke Petrus Hendrikus Leenen

**Affiliations:** Department of Trauma Surgery, University Medical Center Utrecht, Suite G04.232, Heidelberglaan 100, 3584 CX Utrecht, The Netherlands

**Keywords:** Acute hypoxic respiratory failure, ARDS, Polytrauma

## Abstract

**Purpose:**

The definition of acute respiratory distress syndrome (ARDS) has often been modified with Berlin criteria being the most recent. ARDS is divided into three categories based on the degree of hypoxemia using PaO_2_/FiO_2_ ratio. Radiological findings are standardized with bilateral diffuse pulmonary infiltrates present on chest imaging. This study investigated whether chest imaging is relevant in diagnosing ARDS in polytrauma patients.

**Methods:**

The 5-year prospective study included consecutive trauma patients admitted to a Level-1 Trauma Center ICU. Demographics, ISS, physiologic parameters, resuscitation parameters, and ARDS data were prospectively collected. Acute hypoxic respiratory failure (AHRF) was categorized as Berlin criteria without bilateral diffuse pulmonary infiltrates on imaging. Data are presented as median (IQR), *p* < 0.05 was considered significant.

**Results:**

267 patients were included. Median age was 45 (26–59) years, 199 (75%) males, ISS was 29 (22–35), 258 (97%) patients had blunt injuries. Thirty-five (13%) patients died. 192 (72%) patients developed AHRF. AHRF patients were older, more often male, had higher ISS, needed more crystalloids and blood products than patients without AHRF. They developed more pulmonary complications, stayed longer on the ventilator, in ICU and in hospital, and died more often. Fifteen (6%) patients developed ARDS. There was no difference in outcome between ARDS and AHRF patients.

**Conclusions:**

Many patients developed AHRF and only a few ARDS. Patients with similar hypoxemia without bilateral diffuse pulmonary infiltrates had comparable outcome as ARDS patients. Chest imaging did not influence the outcome. Large-scale multicenter validation of ARDS criteria is warranted to investigate whether diffuse bilateral pulmonary infiltrates on chest imaging could be omitted as a mandatory part of the definition of ARDS in polytrauma patients.

**Electronic supplementary material:**

The online version of this article (10.1007/s00068-019-01204-3) contains supplementary material, which is available to authorized users.

## Introduction

Acute respiratory distress syndrome (ARDS) is clinically characterized by severe dyspnea, cyanosis refractory to oxygen therapy, loss of lung compliance, and diffuse alveolar infiltrates on chest radiograph [1]. This is caused by an increased permeability of the alveolar–capillary barrier resulting in lung edema with protein-rich fluid causing an impairment of arterial oxygenation. Lung edema, endothelial and epithelial injuries are accompanied by an influx of neutrophils into the interstitium and broncho-alveolar space. Activation and recruitment of neutrophils play an important role in progression of ARDS [2].

Over the years, the definition of ARDS has often been modified with Berlin criteria being the most recent [3, 4]. To facilitate prognosis, ARDS was divided into three categories based on the degree of hypoxemia using the partial pressure of arterial oxygen and fraction of inspired oxygen (PaO_2_/FiO_2_). Additionally, radiological findings were standardized and bilateral diffuse pulmonary infiltrates on chest X-ray or CT scan (without evidence of heart failure) have to be present [4–6]. Like previous definitions, the Berlin ARDS criteria also have been criticized, and several authors have questioned its usefulness [7–9].

Historically, ARDS has been a significant cause of trauma-related morbidity and mortality, with reported mortality rates up to 40% [10, 11]. Many treatment strategies have been created; however, most of them had limited success [12]. Nowadays, the treatment is still limited and mainly consists of supportive mechanical ventilation with low tidal volume and inspiratory pressure ventilation. With improvement of trauma, critical-care mortality caused by ARDS has decreased in the last years [5, 13–18]. However, it still uses significant intensive care unit (ICU) resources.

In contrast to several studies reporting continuously high ARDS-related deaths [5, 13–17, 19], we recently showed low incidence of ARDS and low mortality rates [18]. However, we did observe many polytrauma patients who developed some degree of acute hypoxic respiratory failure (AHRF) without the bilateral diffuse pulmonary infiltrates that characterize ARDS. We felt that these AHRF patients were similar to ARDS patients except for the bilateral diffuse pulmonary infiltrates. Therefore, we conducted a prospective study in polytrauma patients in which we compared outcome parameters of ARDS patients with similar degree of hypoxic respiratory failure, but without the classical radiological findings (AHRF patients). We hypothesized that there was no difference in outcome between both groups. Further, we hypothesized that chest imaging was only relevant to detect abnormalities that need additional treatment other than supportive mechanical ventilation with low tidal volume and inspiratory pressure ventilation.

## Methods

### Study setting

The study was conducted at an urban major (Level-1) trauma center. From November 2013, a 5-year prospective population-based cohort study was undertaken including all consecutive trauma patients who were admitted to the intensive care unit (ICU) of the University Medical Center Utrecht. This major trauma center is the only Level-1 trauma center in the province of Utrecht and covers the central region of the Netherlands with a relatively small, but densely populated service area of 1500 km^2^ and approximately 1.3 million residents. The service area for neurosurgery facilitates 2.1 million residents. Around 1300 trauma patients with full activation of a trauma team are annually admitted. Approximately, 375 of them are multiply injured (ISS > 15) [20]. Patients included in the study were all admitted to ICU either directly from the emergency department (ED) or postoperatively after urgent surgery was performed. Patients < 15 years of age, isolated injuries caused by asphyxiation, drowning, burns or isolated traumatic brain injury (TBI) were excluded. Patients who died within 48 h were excluded as well.

### Data collection

All data were prospectively collected and included patient demographics, injury severity score (ISS), shock and resuscitation parameters. Admission arterial blood gas analysis, coagulation status, and temperature measurement were performed during resuscitation in ED as part of standard procedures. Arterial blood gas analysis and temperature measurement were repeated on arrival in ICU. Urinary output was measured in the first hour after arrival in ICU. Blood product (packed red blood cells (PRBC), fresh frozen plasma (FFP), and platelets (PLT)) use was recorded in the first 24 h following admission. The Denver multiple organ failure (MOF) scores and ARDS Berlin criteria were registered daily up until 28 days or discharge from ICU. Chest X-rays were performed on a daily basis while in ICU. If necessary, additional CTs of chest were performed. Chest X-rays were selected for review 2 days before and after the development of worst hypoxia. All imaging studies were reviewed by the radiologist on call who was blinded to the pulmonary condition. Primary outcome was development of AHRF or ARDS. Secondary outcomes were mortality, pulmonary complications (pneumonia, pulmonary embolism, thorax empyema), ventilator days, ICU length of stay (ICU-LOS), in-hospital length of stay (H-LOS), and multiple organ dysfunction syndrome (MODS).

### Definitions

ARDS was defined by the Berlin criteria including bilateral diffuse pulmonary infiltrates on chest X-ray or CT scan (without evidence of heart failure); there are three categories of ARDS based on degree of hypoxemia: grade 1 mild (200 < PaO_2_/FIO_2_ ≤ 300), grade 2 moderate (100 < PaO_2_/FIO_2_ ≤ 200), and grade 3 severe (PaO_2_/FIO_2_ ≤ 100), all with positive end expiratory pressure (PEEP) ≥ 5cmH_2_0 [14]. Worst PaO_2_/FIO_2_ ratios were calculated on a daily basis starting from day 1 after trauma.

Acute hypoxic respiratory failure (AHRF); hypoxemia divided in three categories similar to ARDS, but without classical bilateral diffuse pulmonary infiltrates on X-ray.

Multiple organ dysfunction syndrome (MODS) was defined by Denver multiple organ failure (MOF) scores of greater than 3, occurring more than 48 h after injury [9]. Denver MOF score was chosen over sequential organ failure assessment (SOFA) to avoid difficulties by including the Glasgow coma scale (GCS) in the organ failure score. GCS can be challenging to obtain in the trauma patient in ICU, because they are often sedated and intubated for extended periods. This could negatively influence the CNS organ failure score [21].

Urgent laparotomy was defined as a laparotomy that was performed in patients who were transported from ED directly (or via CT scan) to the operating room (OR).

Pneumonia was defined as the development of purulent sputum (with positive cultures of sputum) in conjunction with radiological evidence of a new or progressive pulmonary infiltrate.

### Ethical approval

The local ethics committee approved this prospective observational study (reference number WAG/mb/16/026664).

### Statistical analysis

Data were analyzed using IBM SPSS Statistics version 25.0 (Armonk, NY, USA). Graphs were prepared with GraphPad Prism version 7.04 (San Diego, CA, USA). Results are presented as median and interquartile range (IQR). Comparison of variables was done using Kruksal–Wallis test or Pearson’s Chi-square test in dichotomous data. Statistical significance was defined as *p* < 0.05.

## Results

**Fig. 1:**
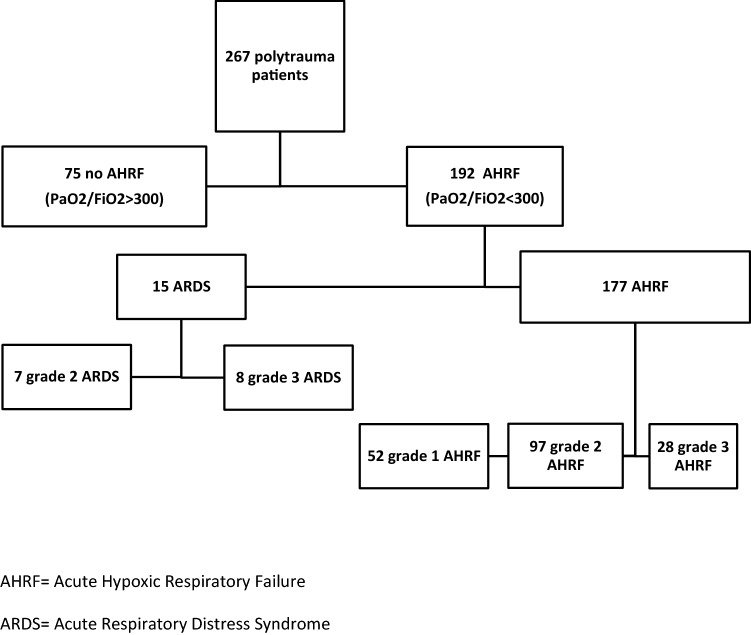
Flowchart of number of patients who developed acute hypoxic respiratory failure and patients who did not

During the 5-year study period, 267 consecutive polytrauma patients who were admitted to ICU and survived 48 h were included. Hundred and thirty-four patients (50%) were intubated pre-hospitally, 72 in ED (27%), 52 in operating room (OR, 20%), 3 in ICU (1%), and 6 were not intubated at all (2%). Hundred and thirty-six (51%) of them were directly admitted to ICU and 131 patients (49%) were transported to the OR for surgery straight from ED and were admitted to ICU postoperatively. Seventy-five percent of the population were male with a median age of 45 (26–59) years. They sustained predominantly blunt injuries (97%) and had a median ISS of 29 (22–35). Even though isolated TBI patients were excluded median AIS head was 3 (1–4). Twenty-four percent of the patients underwent an urgent laparotomy and 83 patients (31%) sustained a pelvic fracture (Table 1). One hundred patients (37%) developed a pulmonary complication (pneumonia, pulmonary embolism or thorax empyema). Patients stayed on the ventilator for 7 (3–12) days. They spent 8 (4–14) days in ICU and 22 (13–33) days in hospital. Eighty-three patients developed MODS (31%, additional data on MODS development, severity, and duration are added as supplemental figures (supplemental Figs. 1 and 2), and 35 patients (13%) died (Table 1). Death in 28 patients was caused by brain injury (80%), 3 patients with high cervical spine injury failed to wean from the ventilator (9%), 1 patient died of MODS (3%), 1 patient died of sepsis (3%), 1 patient due to cardiac arrest (3%), and 1 polytrauma patient died of ARDS (3%) with additional fresh-water submersion. Fifteen patients (6%) developed ARDS (7 patients developed grade 2 ARDS and 8 patients developed grade 3 ARDS). Seventy-five (28%) patients did not develop any signs of acute hypoxic respiratory failure (AHRF). The remaining 192 patients all developed some degree of AHRF; 52 Patients developed grade 1 AHRF, 97 patients developed grade 2 AHRF, and 28 patients developed grade 3 AHRF (Fig. 1). Patients who developed grade 1 AHRF were excluded from further analysis.

**Table 1:** Patient demographics, resuscitation and outcome parameters

	Total cohort (*n* = 267)	No AHRF (*n* = 75)	Gr 2 and 3 AHRF (*n* = 140)	*p* value
*Demographics*				
Age (years)	45 (26–59)	36 (23–55)	50 (33–62)	0.002*
Gender (% male)	199 (75)	47 (63)	114 (81)	0.003*
MOI (% blunt)	258 (97)	71 (95)	138 (99)	0.19
ISS	29 (22–35)	24 (19–29)	29 (24–38)	< 0.001*
AIS head	3 (1–4)	3 (0–4)	3 (2–4)	0.20
AIS face	0 (0–2)	0 (0–2)	0 (0–2)	0.94
AIS chest	3 (2–4)	3 (2–3)	3 (3–4)	0.004*
AIS abdomen	0 (0–3)	0 (0–2)	0 (0–3)	0.81
AIS extremities/pelvis	2 (1–3)	2 (2–3)	2 (1–3)	0.84
AIS external	0 (0–1)	0 (2–3)	0 (0–1)	0.57
Pelvic fracture	83 (31)	25 (33)	45 (32)	0.88
Urgent laparotomy	65 (24)	16 (21)	36 (26)	0.51
*Resuscitation*				
Crystalloids (L)				
< 8 h	4.7 (2.5–6.2)	4.2 (1.3–5.5)	5.1 (2.7–7.3)	0.002*
< 24 h	7.4 (5.3–10.4)	6.0 (3.3–9.5)	8.2 (6.1–11.4)	< 0.001*
PRBC (u)				
< 8 h	1 (0–4)	0 (0–3)	2 (0–5)	0.01*
< 24 h	1 (0–6)	0 (0–3)	2 (0–6)	0.02*
FFP (u)				
< 8 h	0 (0–4)	0 (0–2)	1 (0–4)	0.002*
< 24 h	0 (0–4)	0 (0–2)	2 (0–6)	< 0.001*
PLT (u)^a^				
< 8 h	0 (0–1)	0 (0–0)	0 (0–1)	0.001*
< 24 h	0 (0–1)	0 (0–0)	0 (0–1)	< 0.001*
Outcome				
Pulmonary complications^b^	100 (37)	12 (16)	71 (51)	< 0.001*
Ventilator days	7 (3–12)	2 (1–3)	10 (7–15)	< 0.001*
ICU-LOS (days)	8 (4–14)	3 (2–4)	12 (8–17)	< 0.001*
H-LOS (days)	22 (13–33)	15 (10–23)	27 (15–38)	< 0.001*
MODS	83 (31)	2 (3)	71 (51)	< 0.001*
Mortality	35 (13)	3 (4)	28 (20)	0.001*

Data are expressed as median (IQR) or absolute numbers (%)

*AHRF* acute hypoxic respiratory failure, *MOI* mechanism of injury, *ISS* injury severity score, *AIS* abbreviated injury scale, *MODS* multiple organ dysfunction syndrome, *LOS* length of stay, *H-LOS* hospital length of stay

*Statistically significant

^a^1 unit of platelet contains five donors

^b^Pulmonary complications consisted of pneumonia, pulmonary embolism, thorax empyema

### No-AHRF vs. AHRF/ARDS patients

Patients who developed grade 2 or 3 AHRF (including those patients who developed ARDS) were older (50 (33–62) vs. 36 years (23–55), *p* = 0.002), more often male (81% vs. 63%, *p* = 0.003) and had a higher ISS (29 (24–38) vs. 24 (19–29), *p* < 0.001) (with higher AIS chest (3 (3–4) vs. 3 (2–3), *p* = 0.004) than patients who did not develop any signs of hypoxic respiratory failure. Further, they received more crystalloids < 8 h (5.1 (2.7–7.3) vs. 4.2L (1.3–5.5), *p* = 0.002) and 24 h (8.2 (6.1–11.4) vs. 6.0L (3.3–9.5), *p* < 0.001), and more blood products 8 h and 24 h (Table 1). Patients who had grade 2 or 3 AHRF developed more often a pulmonary complication (51% vs. 16%, *p* < 0.001) than patients without AHRF. Patients who developed grade 2 or 3 AHRF stayed longer on the ventilator (10 (7–15) vs. 2 (1–3) days, *p* < 0.001), longer in ICU (12 (8–17) vs. 3 (2–4) days, *p* < 0.001) and in hospital (27 (15–38) vs. 15 (10–23) days, *p* < 0.001) than no-AHRF patients. They developed more often MODS (51% vs. 3%, *p* < 0.001) and died more often (20% vs. 4%, *p* = 0.001, Table 1).

Patients who later developed AHRF/ARDS had lower PaO_2_ levels, lower pH, and lower saturation both on arrival in ED and ICU, lower blood pressure and higher PaCO_2_ in ED (Table 2). In ED, base deficit (BD) was similar between both groups, whereas on arrival in ICU, BD was lower in patients who later developed AHRF/ARDS (Table 2).

**Table 2:** ED and ICU parameters comparing patients who developed AHRF and patients who developed no acute hypoxic respiratory failure

	No AHRF (*n* = 75)	Gr 2 and AHRF (*n* = 140)	*p* value
ED parameters			
SBP (mmHg)	128 (110–140)	115 (93–136)	0.02*
DBP (mmHg)	80 (67–90)	73 (57–86)	0.04*
Temperature (℃)	35.5 (34.6–36.5)	35.2 (34.3–36.5)	0.53
Hb (mmol/L)	8.2 (7.6–9.1)	8.0 (7.2–8.9)	0.06
Leukocytes (×10^9^/L)	15.7 (11.6–19.6)	15.7 (11.2–20.8)	0.94
Platelets (×10^9^/L)	244 (203–291)	231 (185–278)	0.05
PT	15.0 (14.0–16.8)	15.7 (14.4–17.6)	0.03*
pH	7.34 (7.29–7.38)	7.29 (7.23–7.36)	0.001*
PaCO_2_ (mmHg)	44 (39–50)	48 (42–54)	0.004*
PaO_2_ (mmHg)	261 (129–366)	173 (89–261)	< 0.001*
BD (mmol/L)	− 2.0 (− 5.3 to 1.0)	− 3.5 (− 6.8 to 0.0)	0.08
Sat (%)	100 (99–100)	99 (95–100)	< 0.001*
ICU parameters			
SBP (mmHg)	122 (112–139)	118 (102–135)	0.08
DBP (mmHg)	68 (57–77)	63 (55–71)	0.05
Temperature (℃)	35.4 (34.6–36.0)	35.5 (34.4–36.0)	0.48
Hb (mmol/L)	7.8 (6.8–8.5)	7.5 (6.6–8.2)	0.10
pH	7.34 (7.32–7.39)	7.32 (7.26–7.37)	0.004*
PaCO_2_ (mmHg)	38 (42–46)	43 (40–48)	0.06
PaO_2_ (mmHg)	169 (133–194)	137 (98–166)	< 0.001*
BD (mmol/L)	− 2.5 (− 5.5 to − 0.5)	− 3.9 (− 6.0 to − 1.8)	0.01*
Sat (%)	99 (98–99)	98 (97–99)	< 0.001*
UO (ml)	123 (80–300)	150 (73–300)	0.94

Data are expressed as median (IQR)

*AHRF* acute hypoxic respiratory failure, *MODS* multiple organ dysfunction syndrome, *SBP* systolic blood pressure, *DBP* diastolic blood pressure, *Hb* hemoglobin, *PT* prothrombin time, *BD* base deficit, *Sat* saturation, *UO* urinary output first hour in ICU

*Statistically significant

**Fig. 2:**
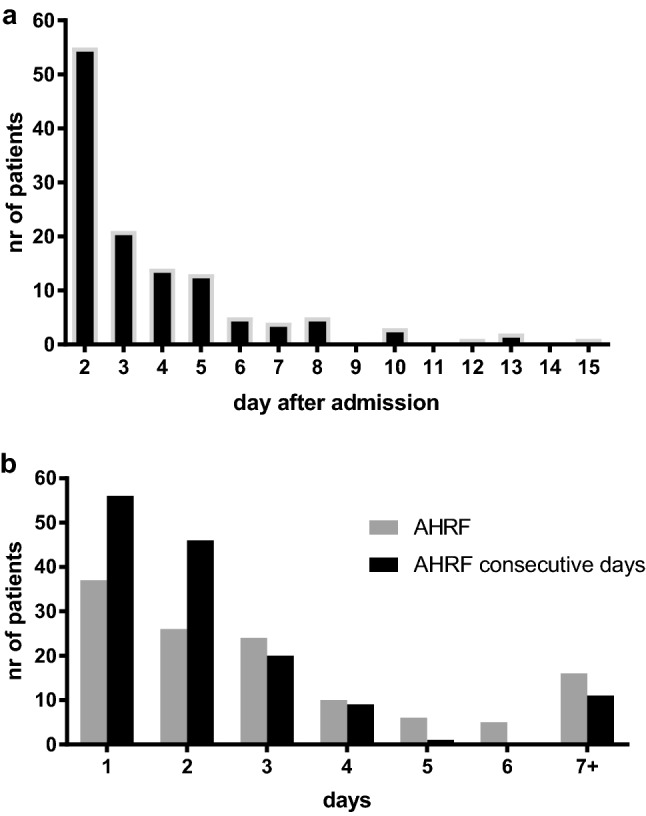
**a** Day of acute hypoxic respiratory failure onset. **b** Duration of acute hypoxic respiratory failure measured in total days (gray) and in consecutive days (black) during admission

Time to AHRF onset was early after trauma; AHRF developed 3 (2–5) days from injury with a length of 2 (1–4) days (Fig. 2a). Twenty-one patients (17%) had AHRF for more than 5 days and 11 patients (9%) had AHRF for more than 5 consecutive days (Fig. 2b). This early onset and short duration of AHRF is comparable to ARDS onset and duration in ARDS patients as we have demonstrated in a previous study [18].

### ARDS vs. AHRF patients

Patients who later developed ARDS had higher PaCO_2_ in ICU (49 (44–56) vs. 43 mmHg (40–47), *p* = 0.004) than patients who developed AHRF but no ARDS. All other parameters such as demographics, injury severity score (ISS), shock and resuscitation parameters, pulmonary complications, and outcome showed no difference between ARDS and AHRF patients.

One of 15 ARDS patients (7%) died of ARDS. He developed severe ARDS 3 days after massive aspiration after fresh-water submersion after a motor vehicle accident with brain injury, maxillofacial, and several cervical spine fractures including dissection of a vertebral artery. He had ARDS for 4 days with lowest PaO_2_/FiO_2_ ratio of 40 and despite venovenous extracorporal life support he died 7 days after admission. Further, two other patients died while having ARDS; one patient suffered from multiple injuries including severe brain injury, and treatment was withdrawn as it was considered as medically futile. The other patient failed to wean from the ventilator after C2 cervical spine injury with myelum contusion resulting in tetraplegia.

One patient with AHRF died of acute hypoxic respiratory failure (3%). He died 45 days after injury after a combination of aspiration and cardiac failure. Thirty-one other patients died while having AHRF; 27 patients died of severe brain injury, 1 of sepsis, 1 of MODS, and 2 patients failed to wean from the ventilator after high cervical spine injury with myelum contusion.

### PaO_2_/FiO_2_ grading related to outcome

**Fig. 3:**
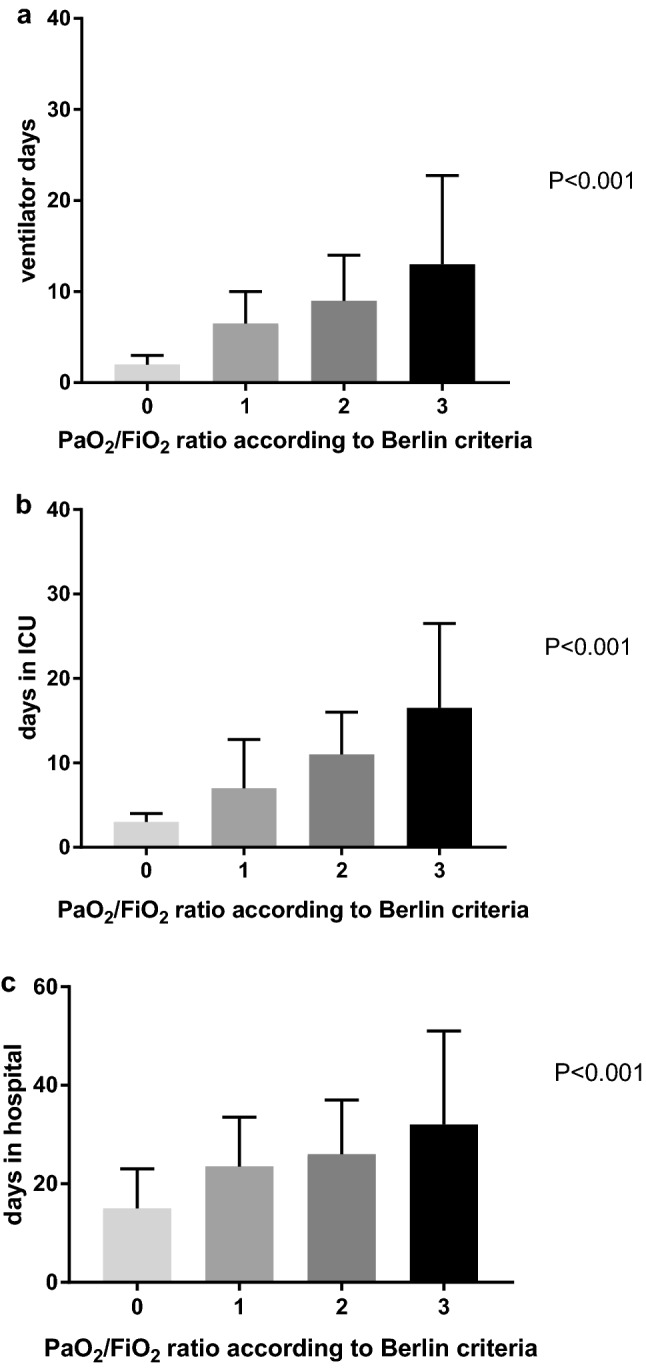
PaO_2_/FiO_2_ ratio related to ventilator days (**a**), days in ICU (**b**), and days in hospital (**c**)

Both ventilator days, days in ICU and in hospital increased with increasing grade of hypoxemia; patients without hypoxemia spent 2 (1–3) days on the ventilator, 3 (2–4) days in ICU and 15 (1–23) days in hospital. Patients with grade 1 hypoxemia spent 7 (4–10) days on the ventilator, 7 (4–13) days in ICU and 24 (15–34) days in hospital. Patients with grade 2 hypoxemia spent 9 (7–14) days on the ventilator, 11 (8–16) days in ICU and 26 (14–37) days in hospital. Patients with grade 3 hypoxemia spent 13 (7–23) days on the ventilator, 17 (12–27) days in ICU and 32 (17–51) days in hospital (Fig. 3). Mortality rates were lowest in grade 0 and 1 hypoxemia (4% and 7%, respectively) and highest in grade 2 hypoxemia (22%) and in grade 3 hypoxemia (14%).

### Relation chest imaging and AHRF

By definition, all ARDS patients had bilateral diffuse infiltrates on chest imaging. Fifty-eight patients (77%) who had no acute hypoxic respiratory failure had normal chest X-rays, whereas 15 patients without AHRF (23%) showed signs of pulmonary contusion or atelectasis on X-ray (Table 3). Hundred and twenty-five AHRF patients (89%) had abnormal chest X-rays, whereas 15 patients (11%) with AHRF had normal X-rays. Interestingly, 13 patients (38%) with pulmonary contusion on chest imaging had no hypoxemia, whereas all patients who developed pneumonia or signs of cardiac failure (bilateral pleural effusion) had some degree of hypoxemia (Table 3). Any discrepancy between the number of pneumonias registered as complication and the number of pneumonias on chest imaging can be explained by the fact that pneumonias, on imaging, were only calculated if present at the time of hypoxia. Sensitivity of chest imaging for diagnosing the cause of the hypoxic respiratory failure was 89%, specificity 77%, positive predictive value 88%, and negative predictive value 79%.

**Table 3:** Degree of hypoxemia related to chest imaging

Finding on chest imaging	No AHRF (PaO_2_/FiO_2_ ratio > 300)	Grade 2 AHRF (< 200 PaO_2_/FiO_2_ ratio > 100)	Grade 3 AHRF (PaO_2_/FiO_2_ ratio < 100)	Total
Normal	58	12	3	73
Hemothorax/pleural effusion	2	34	5	41
Pneumonia	0	8	9	17
Bilateral diffuse infiltrates (ARDS)	0	7	8	15
Lung contusion	13	19	2	34
Atelectasis	2	17	5	24
Cardiac failure	0	7	4	11
Total	75	104	36	215

## Discussion

In this population of severely injured patients, 72% of patients developed some degree of AHRF. These AHRF patients were older, more often male, had higher ISS, needed more crystalloids and blood products than patients who did not develop AHRF. Further, they developed more often pulmonary complications, stayed longer on the ventilator, longer in ICU and in hospital, and died more often. Most patients died of brain injury even though we excluded patients with isolated TBI. The percentage of brain/spinal cord injury-related deaths was higher than most reports about the cause of death in polytrauma [22, 23]. We have shown this in previous studies [18, 24], and this could be partly explained by the fact that our level-1 trauma center is the only referral center for brain and spinal cord injuries in the state [20].

In contrast to high incidence of AHRF, the incidence of ARDS was low (6%). When comparing AHRF patients with ARDS patients, only PaCO_2_ in ICU was different, with ARDS patients having a higher PaCO_2_. All other parameters including resuscitation and outcome were comparable. In other words, the only other difference between ARDS and AHRF patients was bilateral infiltrates on chest X-ray since this is part of the definition of ARDS. One could only speculate why ARDS patients had higher first-measured PaCO_2_ in ICU; is it because the influx of neutrophils into the lung has already started and ARDS is already in progress? Or do these patients develop ARDS, because they did not receive optimal low tidal volume and inspiratory pressure ventilation from the start?

Another interesting finding was that 11% of patients who developed grade 2 or 3 AHRF did not have any abnormalities on their chest X-ray at the time of hypoxia. Further, AHRF developed early (3 days) after injury. Since early AHRF may be related to fluid overload rather than complications such as pneumonia, a possible explanation could be that early pulmonary edema was already causing hypoxia, but was insufficient to be seen on X-ray.

We feel that it would be more useful to use criteria of hypoxemia without the bilateral infiltrates on chest imaging. The fact that a patient is identified as having ARDS rather than AHRF in general does not influence ventilator settings since lung-protective ventilation nowadays is the standard of care anyway. In this study, comparison between patients with and without AHRF showed that AHRF alone has the discriminative power to separate sick from the not-so-sick patients. Another argument for dismissing imaging out of the definition is the fact that many trauma patients sustain pulmonary contusions. Early after injury, it is very difficult to differentiate between bilateral infiltrates caused by ARDS or by bilateral pulmonary contusion. Vice versa, there is also the potential danger of labeling a patient as having ARDS based upon bilateral infiltrates which originate from a different source (such as pulmonary contusions). Obviously, chest imaging itself is still very valuable to diagnose entities such as pneumothorax and hemothorax that need additional treatment to lung-protective ventilation. We, therefore, do not suggest to reduce the use or abandonment of chest imaging in the trauma patients.

Further, a recent study showed that inter-observer reliability of diagnosing ARDS was only moderate (kappa 0.50). The disagreement between clinicians was explained by differences in how chest imaging studies were interpreted [9]. Further, Chung et al. have demonstrated that CT findings cannot differentiate between pathology-proven diffuse alveolar damage as can be seen in ARDS and histopathological features of pneumonia [25].

Like others [4, 5, 19, 26], we have also shown that ventilator days, days in ICU and in hospital increased with increasing grade of hypoxemia. The question is whether grading of hypoxemia according to the Berlin criteria is really relevant. It might be helpful for predicting prognosis, but not for diagnosis of ARDS since treatment strategies are limited and not reserved for a specific grade of hypoxemia. This has also been previously addressed by others [6]. Also, both autopsy study [27] and biopsy study [28] demonstrated that the Berlin criteria did not correlate with the presence of diffuse alveolar damage in more than 50% of patients categorized as moderate or severe ARDS.

These arguments raise the question whether the definition of ARDS is specific enough. Although the definition of ARDS, which is based on consensus criteria, is useful for screening, it is less suitable as a diagnostic test. ARDS is a heterogeneous syndrome and it is unlikely that it will be amenable to a single intervention. If consensus definitions of a heterogeneous syndrome are not specific enough, patient selection for interventional trials could be incorrect resulting in false-negative results. This issue has also been addressed by Laffey and Kavanagh in their comments on many negative trials in critical care [29].

AHRF patients received more crystalloids < 8 h and < 24 h after admission than no-AHRF patients; however, it was noted that crystalloid infusion was high in all the included patients. During the first 24 h after admission most patients were in ICU where intensivists decided on resuscitation, since there is a closed-format ICU in our hospital. During the beginning of the studies, there was an ongoing debate between trauma surgeons and intensivists on resuscitation in trauma patients. However, in recent years, there has been an increased awareness of the detrimental effects of excessive amount of crystalloids in severely injured patients. Nowadays, hemostatic resuscitation is aimed for in all polytrauma patients.

One of the limitations of this study is that it was conducted in polytrauma patients only. ARDS is a syndrome that occurs in a wide spectrum of patients of whom the majority are not trauma patients. In some patient groups, other than trauma, the finding of bilateral pulmonary infiltrates is relevant for diagnostic and prognostic purposes. We acknowledge that our data only address a small subset of patients suffering from ARDS.

Further, this study was performed at a single institution in which the clinical treatment and research were conducted by the same clinicians. Patients with isolated head injuries were excluded, because of possible different physiologic response to trauma, although we did include polytrauma patients who had associated head injuries. We chose to include these patients, since many of our severely injured patients have associated brain injury and they are also prone to ARDS. Another limitation is that we neither prospectively collected data on tidal volume and plateau pressures nor did we collect data on comorbidities that could have contributed to hypoxia such as COPD or pre-existing heart failure.

## Conclusions

In conclusion, in this polytrauma population, many patients developed AHRF and only a few ARDS. Chest imaging did not seem to influence treatment strategies and outcome, since patients with similar hypoxemia but without bilateral diffuse pulmonary infiltrates had similar outcome as ARDS patients. Large-scale multicenter validation of ARDS criteria is warranted to investigate whether diffuse bilateral pulmonary infiltrates on chest imaging could be omitted as a mandatory part of the definition of ARDS in polytrauma patients.

## Electronic supplementary material

Below is the link to the electronic supplementary material.

Supplemental Fig. 1. A. Day of onset of Multiple Organ Dysfunction Syndrome. B. Duration of Multiple Organ Dysfunction Syndrome * some patients had several periods of MODS (EPS 18 kb)

Supplemental Fig. 2. A. Individual Denver MOF scores of patients who developed Multiple Organ Dysfunction Syndrome expressed per grade of organ failure and per category of organ failure. B.Order of failing organs related to days after admission (EPS 34 kb)

## Data Availability

The datasets used and/or analyzed during the current study are available from the corresponding author on reasonable request.
